# DENV-Mimetic Polymersome Nanoparticles Bearing Multi-Epitope Lipopeptides Antigen as the Next-Generation Dengue Vaccine

**DOI:** 10.3390/pharmaceutics14010156

**Published:** 2022-01-10

**Authors:** Nur Adilah Abdul Rahman, Abdin Shakirin Mohamad Norpi, Muhammad Luqman Nordin, Mohd Cairul Iqbal Mohd Amin, Abdullah Al-Hadi Ahmad Fuaad, Nor Azila Muhammad Azami, Nirmal Marasini, Fazren Azmi

**Affiliations:** 1 Centre for Drug Delivery Technology, Faculty of Pharmacy, Universiti Kebangsaan Malaysia, Jalan Raja Muda Abdul Aziz, Kuala Lumpur 50300, Malaysia; dila.nr97@gmail.com (N.A.A.R.); abdin22shah@gmail.com (A.S.M.N.); luqman.n@umk.edu.my (M.L.N.); mciamin@ukm.edu.my (M.C.I.M.A.); 2 Faculty of Pharmacy and Health Sciences, Royal College of Medicine Perak, Universiti Kuala Lumpur, No. 3 Jalan Greentown, Ipoh 30450, Malaysia; 3 Faculty of Veterinary Medicine, Universiti Malaysia Kelantan, Pengkalan Chepa, Kota Bharu 16100, Malaysia; 4 Department of Chemistry, Faculty of Science, University Malaya, Kuala Lumpur 50603, Malaysia; hadifuaad@um.edu.my; 5 UKM Medical Molecular Biology Institute, University Kebangsaan Malaysia, Jalan Ya’acob Latiff, Bandar Tun Razak, Cheras, Kuala Lumpur 56000, Malaysia; azila_azami@ukm.edu.my; 6 Faculty of Medicine, School of Biomedical Science, The University of Queensland, St. Lucia, QLD 4072, Australia; n.marasini@uq.edu.au

**Keywords:** multi-epitope, peptide vaccine, nanoparticles, dengue virus, polymersome

## Abstract

Dengue remains a severe threat to public health. The safety and efficacy of the licensed dengue vaccine is not clinically satisfactory, which necessitate the need of new approach in designing an effective dengue vaccine without eliciting adverse reaction. Herein, we have designed a lipidated multi-epitope peptide vaccine (LipoDV) that can elicit highly targeted humoral and cell-mediated immune responses. To improve its immunogenicity, LipoDV was presented on the surface of MPLA-functionalized polymersome nanoparticles (PNs-LipoDV-MPLA). The as-constructed vaccine delivery platform resembles the structural morphology of DENV owing to its spherical nanoscale particle size and surface immunostimulatory properties given by LipoDV and MPLA that emulating the functional role of DENV E and prM/M proteins respectively. A proof-of-concept study demonstrated that BALB/c mice immunized with PNs-LipoDV-MPLA induced a stronger antigen-specific antibody response with an enhanced cell-mediated immunity as characterized by the elevated IFN-γ secretion in comparison to other tested vaccine candidates which possess a lesser structural trait of DENV. The DENV-mimicking nanoparticles vaccine exhibited negligible toxicity as analyzed by hemolytic test, MTT assay, histopathological examination and abnormal toxicity test on immunized mice. Collectively, our study provides a strong foundation in designing an effective peptide-based vaccine delivery platform against DENV infection.

## 1. Introduction

Dengue is an arthropod-borne disease that is transmitted to humans through the bites of female *Aedes aegypti* or *Aedes albopictus* mosquitos which have been infected with a dengue virus (DENV). The DENV can be classified into four genetically related but antigenically distinct serotypes, designated as DENV 1–4. Infection by all dengue serotypes causes a wide spectrum of diseases, ranged from asymptomatic/mild febrile illness to severe clinical manifestations that can lead to life-threatening dengue hemorrhagic fever (DHF) and dengue shock syndrome (DSS) [1]. Dengue has been declared to be endemic in over 100 countries (primarily in the tropical and subtropical regions) and continue to be a global threat to public health. It has been estimated that 390 million dengue infections occur annually across the world and contribute to approximately 25,000 casualties [2]. Dengue cases remain prevalent and high in the tropical and subtropical regions, predominantly in Southeast Asia [3]. Currently, there is no antiviral drug for dengue treatment. The current preventive measures against dengue are directed towards vector control, which offer a temporary solution.

Vaccination remains the most effective way for long-term prevention and control of dengue infections. The first licensed dengue vaccine, Dengvaxia^®^ (CYD-TDV) was first introduced into the clinical setting in December 2015. The vaccine efficacy and safety profile, however, varied according to the vaccination recipient’s serostatus and age. The use of CYD-TDV, a live attenuated virus (LAVs)-based vaccine had an increased risk of clinically severe dengue in seronegative (those who were without prior dengue infection) vaccinated individuals. Notably, a higher risk of hospitalization was observed among children ages between 2–5 years [4]. This unprecedented situation that hampers the use of CYD-TDV has been associated with a phenomenon called antibody-dependent enhancement (ADE) [5]. ADE occurs when the raised antibodies were insufficient to completely neutralize the virus, and these sub-neutralizing antibodies instead facilitate the virus entry into host cells, which subsequently enhanced viral loads and disease severity. The use of LAV as vaccine antigen was not favorable as a natural immune response against DENV viruses was found to be predominantly directed against membrane protein (prM/M), which cross-reacted among DENV serotypes at suboptimal neutralizing effect, thus suggesting its involvement in ADE induction [6].

**Figure 1:**
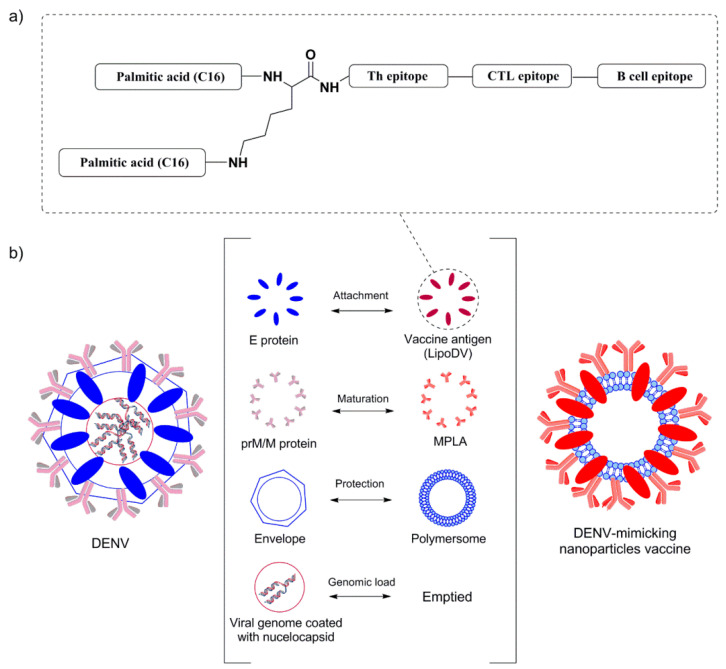
(**a**) A schematic diagram of the LipoDV as a dengue vaccine antigen. LipoDV featured a linear polypeptide consisting of B cell (E_383−397aa_: EPGQLKLNWFKKGSS), T cell (E_345−359aa_: RHVLGRLITVNPIVT) and Th (E_352−368aa_: LITVNPIVTEKDSPVNIE) epitopes bearing two copies of palmitic acid (C16) attached through the N-terminus and the sidechain of lysine. (**b**) Idealized structural representation of the DENV-mimicking nanoparticle platform technology.

As such, a newer generation of tetravalent dengue vaccine focusing on inducing a specific type of immune response is an emerging strategy to minimize the risk of ADE development. Peptide-based vaccines appear as suitable candidates for dengue vaccine development as they can elicit epitope-specific antibodies and immune responses, thus eliminating the risk of vaccine-associated adverse effects, such as autoimmune and/or allergenic reactions. In addition to antibody responses, cell-mediated immunity has a profound role in conferring protection against DENV. T cell epitopes are responsible for the generation of long-lasting cellular immunity capable of eradicating circulating viruses and virus-infected cells [7]. It has been evidenced that DENV infection in a group of mice with cytotoxic T cells (CTLs) depletion reduced the mice’s ability to clear DENV and resulted in considerably greater viral loads in the serum, spleen and brain compared to undepleted mice [8]. On the other hand, the activation of T helper (Th) cells is essential for effective vaccination as it plays a central role in maintaining and priming both effector CTLs and B cells function in promoting memory immune responses [9]. In pursuit of these immunological rationales, we have designed a multi-epitope peptide dengue vaccine that can induce the activation of CTLs, Th and B cells. The respective antigenic B cell (E_383−397aa_) and T cell (E_345−359aa_) peptide epitopes were derived from the domain III region of the DENV-2 E protein (EDIII) (NCG strain) [10]. The known Th (E_352−368aa_) peptide epitope was derived from DENV-2 EDIII of Jamaica strain and has been previously reported to enhance the neutralizing antibody response that cross-reacted against DENV serotypes [11]. EDIII is known as the prime target of highly potent DENV neutralizing antibodies as it possesses a receptor-binding region [12]. Thus, cellular and humoral immunity directed towards the EDIII region can confer protection against DENV with minimal risk of ADE development. The multi-epitope polypeptide was further conjugated to two copies of palmitic acid (C16) by using lysine residue as the branching core at the N-terminal, named LipoDV (Figure 1a). Peptide lipidation is a prominent strategy to improve peptide’s immunogenicity due to the recognition of lipid tail by toll-like receptor 2 (TLR2) [13,14]. 

Peptide vaccines represent an alternative strategy for a safer dengue vaccine development due to their minimalistic antigenic composition. However, the removal of ‘danger signal’ components that are typically possessed by a pathogen reduce the prospective recognition by immune cells which relatively lead to poor efficacy of peptide vaccine. Thus, the use of a vaccine delivery system incorporated with potent immunostimulants is necessary to trigger the desired immune response against the peptide antigen. Herein, we reported the development of a novel multifunctional nanoparticle platform that emulated the external structural and functional traits of a DENV for a safe and efficient presentation of the peptide antigen to the immune system (Figure 1b). DENV virion structure is characterized as spherical nanoparticles. The core of DENV consists of viral genome-nucleocapsid complexes that are surrounded by a well-organized lipid bilayer membrane, the viral envelope. The surface of the viral envelop is embedded by the prM/M and E proteins that are critical for the maturation and attachment of DENV particles to host cells, respectively. In this DENV-mimicking platform, the virus envelope was mimicked by the self-assembled polymersome made up of amphiphilic di-block copolymer poly(butadiene)-poly(ethylene oxide) (PBD-PEO). Due to the increased bilayer thickness, polymersomes are generally more chemically stable and less permeable than conventional liposomes [15]. The prM/M functionality was emulated by the incorporation of monophosphoryl-lipid A (MPLA) while the E protein was resembled by the constructed lipidated antigen, LipoDV. MPLA is a well-known TLR4 agonist which significantly induces the maturation of antigen presenting cells for efficient processing of vaccine antigen [16]. The two lipid tails anchored by LipoDV recapitulated the functionalities of protein E in inducing host membrane cells permeation and cell receptor binding. The key physicochemical properties such as size, morphology and surface charge of the fabricated nanoparticles were characterized. The capacity of the nanoparticle-based vaccine carrier to be taken up by APCs was evaluated in vitro. Finally, to address our question of whether the formulated DENV-mimicry nanocarrier platform would serve as a potent vaccine delivery system, several dengue vaccine candidates; LipoDV alone and its encapsulation within lipophilic bilayer of polymersome with (PNs-LipoDV-MPLA)/without (PNs-LipoDV) the presence of MPLA were assessed for their safety and immunogenicity effects in mice, particularly in inducing humoral and cell-mediated immune responses. The structure-activity relationship analysis revealed that dengue vaccine candidate, PNs-LipoDV-MPLA which was closely resembled DENV structural morphology induced strong antibody responses that were regulated by a balanced Th1/Th2 polarization and exhibited a satisfactory safety profile. 

## 2. Materials and Methods

### 2.1. Materials

All chemicals and reagents were at the highest analytical grade and were used directly without any further purification unless otherwise stated. PBD_32_-PEO_21_ di-block copolymers were obtained from Polymer Source (Montreal, QC, Canada). Dimethyl sulfoxide (DMSO), thiazolyl blue tetrazolium bromide (MTT), phosphate buffer saline (PBS), Triton^TM^ X-100, MES buffer, lysis buffer and anti-Mouse IgG conjugated to horseradish peroxidase were purchased from Sigma-Aldrich (Saint Louis, MO, USA). Eagle’s Minimum Essential Medium (EMEM) and IMDM Glutamax medium Fetal Bovine Serum (FBS) were purchased from Addexbio (San Diego, CA, USA). Fetal Bovine Serum (FBS) and penicillin/streptomycin were purchased from Gibco (Jenks, OK, USA). 2-mercaptoethanol (50 µM) and SnakeSkin^TM^ dialysis tubing (7K MWCO) were supplied by Thermo Fisher Scientific (Waltham, MA, USA). CD11c was purchased from eBioscience (San Diego, CA, USA), while F4/80 was obtained from BioLegend, Pacific, (San Diego, CA, USA).

### 2.2. Synthetic Peptide 

LipoDV(C16K(C16)LITVNPIVTEKSPVNIERHVLGRLITVNPIVTEPGQLKLNWFKKGSS-NH_2_) and B cell peptide epitope (E_383−397aa_: EPGQLKLNWFKKGSS) were synthesized and purified by Bankpeptide Co., Ltd. (Hefei, China). The chemical identity of the peptides was confirmed by the observed molecular weight (LipoDV: MW 5956.25 g/mol and E_383−397aa_: MW 1718.90 g/mol) as determined by ESI-MS. Both peptides were obtained at a purity greater than 95% as measured by analytical RP-HPLC based on the area under the curve analysis.

### 2.3. Polymersome-Based Nanoparticle Vaccine Formulation

The polymersome nanoparticles (PNs) vaccine were prepared via film rehydration method with slight modifications [17]. Briefly, 3.0 mg of PBD_32_-PEO_21_ diblock copolymer was dissolved in 0.6 mL of chloroform (CHCl3) in a round bottom flask. LipoDV (1 mg in 1 mL acetonitrile) was added to the above polymer solution to prepare LipoDV-loaded PNs (PNs-LipoDV). The solvent mixture was evaporated under reduced pressure to remove the organic phase at 37 °C until a thin polymer film formed on the wall of the flask and dried overnight in a desiccator. Then, the polymer film was rehydrated with 600 µL prewarmed sterile Milli-Q water and stirred at 25 °C. The resulted cloudy dispersion was extruded using a mini extruder (Avanti^®^ Mini-Extruder, Avanti Polar Lipid, Alabaster, AL, USA) through a 400 nm polycarbonate membrane for 20 times in both directions followed by another series of 20 times extrusion using a 100 nm polycarbonate membrane. The formulation of PNs loaded with both LipoDV and MPLA (PNs-LipoDV-MPLA) was prepared in a similar protocol to PNs-LipoDV, with the only difference was being that 3 µg of MPLA was added dropwise to the PBD_32_-PEO_21_ suspension before LipoDV was added. The concentration of MPLA was finalized when the particle surface charge reached the maximum negative value of zeta potential (fully saturated).

### 2.4. Physiochemical Characterization 

The formulated nanoparticles vaccines were characterized for hydrodynamic particle size, polydispersity index (PDI) and zeta potential via dynamic light scattering technique at a backscattering angle of 173° at 25 °C in folded capillary cuvettes by a using Malvern Zetasizer Nano ZS (Malvern, UK). The obtained data were processed using Malvern’s Zetasizer software 6.2 and expressed as an average of at least three measurements for each batch. Zeta potential and particle size values were also used as the indication of complete coverage of the nanoparticle’s functionalization with MPLA.

### 2.5. Morphological Analysis

The morphology of the nanoparticles vaccines was characterized by using a Talos L120C Transmission Electron Microscope (TEM, Thermo Fisher, Waltham, MA, USA) at an accelerating voltage of 100 kV. In brief, a drop of diluted nanoparticles solution was placed in a 200-mesh carbon-coated grid for 5 min. Upon particles settlement in the grid, negative staining was performed using 2.5% uranyl acetate before capturing the microscopic image.

### 2.6. Encapsulation Efficiencies 

The encapsulation efficiency (EE) of vaccine antigen, LipoDV inside the nanoparticles was measured by using the dialysis method. Briefly, nanoparticles solution was added to a dialysis tubing (SnakeSkin^TM^) of a molecular weight cut-off = 7 kDa. The sample (containing free LipoDV) was collected after 2 h of dialysis. The standard calibration curve of LipoDV (20–120 µg/mL) was plotted and the quantification of the loaded LipoDV was carried out by UV-vis spectroscopy at 280 nm. The percentage of EE was calculated using the following formula:

EE=(Total LipoDV added− Free LipoDV in buffer/Total LipoDV  added)×100%

### 2.7. Ex Vivo Hemolysis Assay 

Hemolysis assay was carried out using fresh human red blood cells as described elsewhere with slight modification [18]. In brief, a fresh human blood sample was obtained and placed in a heparinized tube to prevent coagulation. The blood sample was centrifuged at 750× *g* for 15 min to extract the serum and washed three times with sterile PBS. The same volume of sterile PBS was added after the serum was removed to produce a 1% suspension of red blood cells. Then, 100 µL of tested compounds were added into the microcentrifuge tube at various concentrations of 1 mg/mL, 0.5 mg/mL and 0.124 mg/mL, along with 100 µL of red blood cells suspension. Triton X-200 with a final concentration of 0.5% (vol/vol) in sterile PBS was used as a positive control while sterile PBS was used as a negative control. The tubes were incubated for 2 h at 37 °C in an S1500 shaker (Stuart Scientific, Nottingham, UK). Then, the tubes were centrifuged at 750× *g* for 15 min after incubation, and 50 µL of supernatant was extracted in triplicates and transferred onto a 96-well plate. The absorbance was measured at 540 nm by using a spectrophotometric microplate reader (NanoQuant Infinite M200 Pro, Tecan, Switzerland). The percentage of hemolysis activity was calculated using the following equation:

Hemolysis, %=(AbsCompound−AbsPositive/AbsNegative − AbsPositive)×100%

AbsCompound represent the value of nanoparticle formulations, AbsPositive represents value of positive control, and AbsNegative represents value of negative control.

### 2.8. MTT Cytotoxic Assay

The in vitro cytotoxicity activity of the constructed PNs vaccines was evaluated against human embryonic kidney cell (HEK293) based on established protocol [15]. Briefly, HEK293 was cultured in EMEM supplemented with 10% FBS and 1% penicillin/streptomycin. Cells were plated in a clear 96-well plate (5.0 × 10^4^ cells/well) and maintained for 24 h at 37 °C with 5% CO_2_ in a humidified chamber for cell attachment. PNs were prepared and serially diluted in DMEM to give concentrations ranging from 0.25 to 1 mg/mL. Then, 200 µL of each diluted solution was added to each wells containing attached cells in triplicate after the used media were removed and incubated with 5% CO_2_ at 37 °C for 48 h. After the incubation, the media were removed and 20 μL of 5 mg/mL MTT solutions in PBS were added to each well and further incubated for 4 h with 5% CO_2_ at 37 °C. The media was then replaced by 100 µL of dimethyl sulfoxide (DMSO) and assiduously mixed to solubilize the formed formazan crystal. DMSO alone was used as a blank and the untreated cells were used as a negative control. The absorbance was measured using NanoQuant infinite M200 Pro spectrophotometric microplate reader (Tecan, Männedorf, Switzerland) at 570 nm and the viability of the cells was calculated using the following formula:

Cell Viability, %=(AbsCompound− AbsBlank/AbsNegative− AbsBlank)×100%

### 2.9. Antigen-Presenting Cell Uptake Assay

This assay was performed based on a previously published protocol with some modifications [19]. Under aseptic conditions, spleens were dissected from female Balb/c mice. The isolated spleens were harvested and homogenized by passing through a cell strainer to dissociate into a single cell suspension. The red blood cells were lysed using erylysis buffer (pH 7.2–7.4, sterilized). The resultant splenocytes were seeded into a 12-well plate (cell density: 2 × 10^5^ cells/well) in phenol-free IMDM Glutamax medium, supplemented with 10% FBS, 50 µM 2-mercaptoethanol, 100 IU/mL penicillin, and 100 µg/mL streptomycin. Then, the tested compounds; PNs-based vaccine candidates were labelled with Dil (1,1 dioctadecyl-3,3,3′,3-tetramethylinocarbocynine perchlorate). The Dil-labelled vaccine compounds at a concentration of 50 μM concentration were then added to the wells and incubated for 24 h. Cells adhering to the plates were scraped and incubated with Fc-block for 20 min at 4 °C. After incubation, the plates were centrifuged and the supernatants were discarded and resuspended in FACS buffer (PBS, 0.02% sodium azide, 0.5% bovine serum albumin (BSA)) containing CD11c and F4/80 antibodies at 4 °C for 30 min. The cells were centrifuged again and then analyzed using the LSR II flow cytometer (BD FacsCanto II, Franklin Lakes, NJ, USA) in a 0.5 mL FACS buffer (PBS, 0.02% sodium azide, 0.5% BSA) (Supplementary Figure S1). The fluorescence intensity of dendritic cells and macrophages that were treated with normal saline was used as a control. The actual uptake was calculated as the percentage of cells for Dil and CD11c or Dil and F4/80.

### 2.10. Mice Immunization Study

All of the mice handling protocols and guidelines were carried out according to University Kebangsaan Malaysia Animal Ethics Committee (UKMAEC, Ref No: FF/2020/FAZREN/25-MAR./1097-APR.-2020-APR.-2021). Female Balb/c mice (6-week-old) were purchased from UKM Laboratory Animal Research Unit (LARU). All mice were maintained under pathogen-free conditions with free access to food and water. Mice were divided into five groups with six mice in each group (n = 6). Each mouse was immunized via subcutaneous injection into the loose skin over the neck region on day 0, followed by two booster doses on day 10 and 20 with different vaccine candidates (free LipoDV, PNs-LipoDV and PNs-LipoDV-MPLA) at a dose of 30 µg of LipoDV immunogen at a volume of 50 µL. Each mouse in the positive control group was immunized with 30 µg of LipoDV emulsified in Complete Freund’s Adjuvant (CFA) to a total volume of 50 µL and boosted with 30 µg of LipoDV in 50 µL of normal saline in similar immunization interval. Negative control mice were immunized with 50 μL of normal saline. 

### 2.11. Serum Collection and Dissection of Selected Organs

Blood samples were collected via tail bleed on days 9 and 19, while blood samples on day 27 were collected by cardiac puncture. Blood was centrifuged at 10,000× *g* for 10 min to separate the serum. The collected serum samples were stored at −80 °C until further analysis. On day 27, mice were euthanized, and the specimens including spleen tissues, liver and kidney were collected under aseptic conditions and fixed in 4% *v*/*v* buffered formalin for preservation until further and were kept at −80 °C for subsequent analysis.

### 2.12. Evaluation of Antigen-Specific Systemic IgG Antibodies

The collected serum samples were used for the analysis of B cell epitope (E_383-397aa_)-specific serum IgG antibodies using an enzyme linked immunosorbent assay (ELISA). The assay was performed using DuoSet ELISA Ancillary Reagent Kit (DY008; R&D Systems, Minneapolis, MN, USA) as previously reported [20]. Briefly, the polycarbonate plates were coated with diluted E_383-397aa_ peptide antigen to 0.5 mg/mL in ELISA coating buffer. Then, the plates were washed using washing buffer before being blocked with 150 μL of blocking buffer (3% blocking buffer in PBS with 0.05% Tween-20). A 1:100 concentration of serum to reagent diluent (0.1% BSA in PBS, with 0.05% Tween-20) was serially diluted 1:2 down the plate and incubated for 2 h at 37 °C. Then secondary antibody (antimouse-IgG conjugated to horseradish peroxidase mixture with hydrogen peroxide and tetramethylbenzidine) was diluted to 1:3000 before being added to the plates. 100 μL of color reagent was added to each well and the plate was incubated for 20 min at room temperature in the dark according to the manufacturer’s instructions. After the incubation, 50 μL of stop solution was added and the plate was read using a spectrophotometer (NanoQuant infinite M200 Pro, Tecan, Männedorf, Switzerland) at 450 nm. The antibody titer was expressed as the lowest dilution that gave an absorbance of >3 standard deviations (SD) above the mean absorbance of negative control wells (contains negative control mouse serum). Statistical analysis (*p* < 0.05 = statistical significance) was determined using one-way ANOVA followed by Turkey post hoc test using GraphPad Prism Software 8.0, (San Diego, CA, USA).

### 2.13. Spleen Cell Culture and Cytokines Quantification

The aseptically dissected spleen tissues on day 27th (7 days after final immunization) were immediately homogenized using a sonicator (Model MSX-Q125220, Qsonica, Newtown, CT, USA) at 25 HZ for 5 min for cytokine secretion analysis without restimulation with antigen. Cells were collected and washed using cold PBS. 50 μL of RIPA buffer was added per 1 mg of tissue to lyse the red blood cells. The supernatants were isolated by centrifugation at 16,000 rpm for 10 min at 4 °C. The supernatants were diluted to 50 mg/protein with RIPA buffer and 150 µL of each sample was inserted onto a round-bottom 96-well plate. The cytokines (IFN-γ, IL-2 and IL-4) expression was identified using commercially available MILLIPLEX^®^ Multiplex Assays Using Luminex^®^ kit (Merck Millipore, Watford, UK) according to the manufacturer’s protocol. Each sample was assayed in triplicate, and the cytokine standards and quality controls were supplied by the manufacturer and standardized on each plate as a referral point.

### 2.14. In Vivo General Toxicity Analysis

The immunized mice were monitored closely throughout the experiment period for any toxicity signs and symptoms. Bodyweight (recorded at every predetermined time interval) and mortality were observed throughout the immunization point.

### 2.15. Histology Analysis

The histological examinations of livers and kidneys of each experimental group were performed based on an established protocol [21]. In brief, the isolated organs were fixed in 10% buffered formalin. Then, the tissue blocks were immersed in paraffin wax and routine sections were stained with hematoxylin and eosin. The hematoxylin-eosin (HE) stained sections were examined by light microscopy on an Olympus BX40-B microscope at a magnification of 40×.

### 2.16. Stability of Polymersome Nanoparticle

The stability of the blank PNs (either with/without the presence of MPLA) was analyzed for seven months under different conditions: 25 °C ± 2 °C (at room temperature), 4 °C (in the refrigerator) and 40 °C (at elevated temperature). The samples were taken in specified intervals for analysis of charge and size.

## 3. Results

### 3.1. Physiochemical Characterization of the Polymersome-Based Nanoparticle Vaccines

The physicochemical properties, including hydrodynamic particle size, size distribution (based on PDI value) and the particle’s surface charge (represented by zeta potential) were characterized for the formulated PNs vaccines; PNs-LipoDV and PNs-LipoDV-MPLA (Table 1). Both formulated PNs exhibited a uniform particle size (PDI value < 0.3) of about 130 nm. The incorporation of MPLA in the polymersome nanoparticles did not change the particle size and dispersion homogeneity. However, PNs-LipoDV-MPLA had a greater negative charge than PNs-LipoDV. This data demonstrated that the inclusion of MPLA enhanced the colloidal stability of the polymersome dispersions in aqueous solution. Furthermore, the increase in the negative value of zeta potential indicated the presence of MPLA on the particle surface due to its negatively charged phosphate group. The encapsulation efficiency of the dengue vaccine antigen, LipoDV was above 93% for both nanoparticles’ formulations.

### 3.2. Structural Morphology

**Figure 2:**
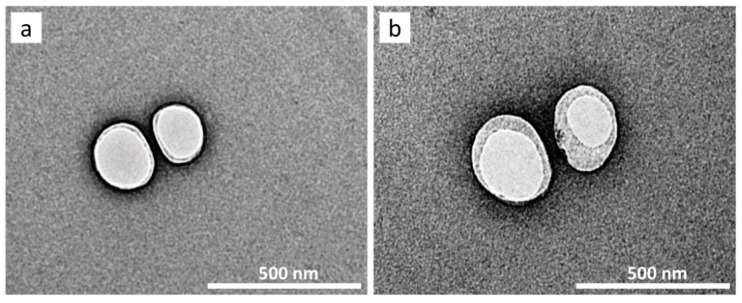
TEM images of (**a**) PNs-LipoDV and (**b**) PNs-LipoDV-MPLA, scale bar 500 nm.

The morphology of the formulated PNs vaccines was studied by using transmission electron microscopy (TEM) and the results were displayed in Figure 2. The TEM images revealed that the amphiphilic PBD_32_-PEO_21_ di-block copolymer was capable to self-assemble into nanoscale particles possessing a vesicular structure analogous to the liposome. The bilayer shell of the di-block copolymer and an aqueous core was visualized in the TEM image. The mean diameter of both unilamellar PNs-LipoDV and PNs-LipoDV-MPLA were in the range of 100–150 nm, which were well coincided with particle size data obtained via DLS. However, PNs-LipoDV-MPLA exhibited a thicker bilayer thickness compared to PNs-LipoDV which implies successful encapsulation of MPLA within the hydrophobic bilayer of the polymer.

### 3.3. Uptake Studies of PNs Vaccine by DC and Macrophages

**Figure 3:**
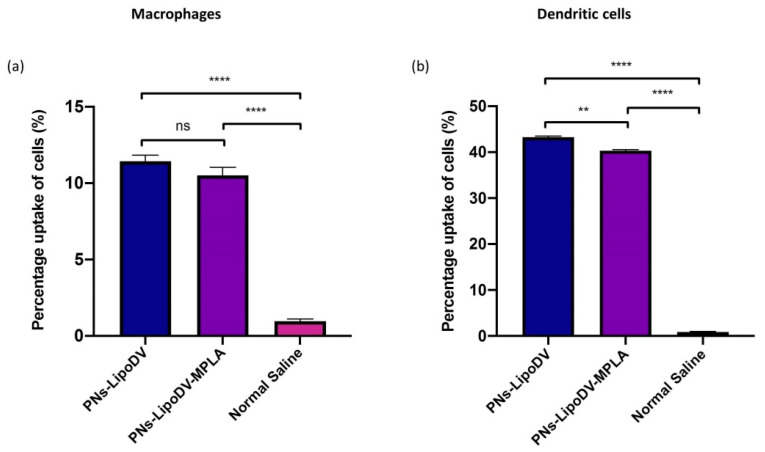
Macrophage (**a**) and dendritic cell (**b**) uptake analysis for PNs-LipoDV, PNs-LipoDV-MPLA and normal saline (negative control). Bars represent the mean and standard deviations of the percentage of cell marker positive for each group. The differences between the groups were analyzed using one-way ANOVA and post hoc Tukey test (ns, *p* > 0.05; ** *p* < 0.01; **** *p* < 0.0001).

The presentation of sufficient amounts of vaccine antigens and immunomodulatory compounds to the APCs, particularly DCs and macrophages is a focal point for orchestration of the adaptive immune response. Thus, we have examined the uptake efficiency of the formulated PNs vaccines using murine CD11c+ DCs and F4/80+ macrophages (Figure 3). Both vaccines; PNs-LipoDV and PNs-LipoDV-MPLA were efficiently (**** *p* < 0.0001 vs. PBS) taken up by APCs. A relatively greater uptake percentage of the nanoparticles was observed in DCs compared to macrophages. There was no significant difference in uptake between PNs-LipoDV and PNs-LipoDV-MPLA by macrophages. Nevertheless, PNs-LipoDV showed significantly higher uptake by DCs in comparison to PNs-LipoDV-MPLA.

### 3.4. In Vitro Cell Viability and Hemolysis Assay 

**Figure 4:**
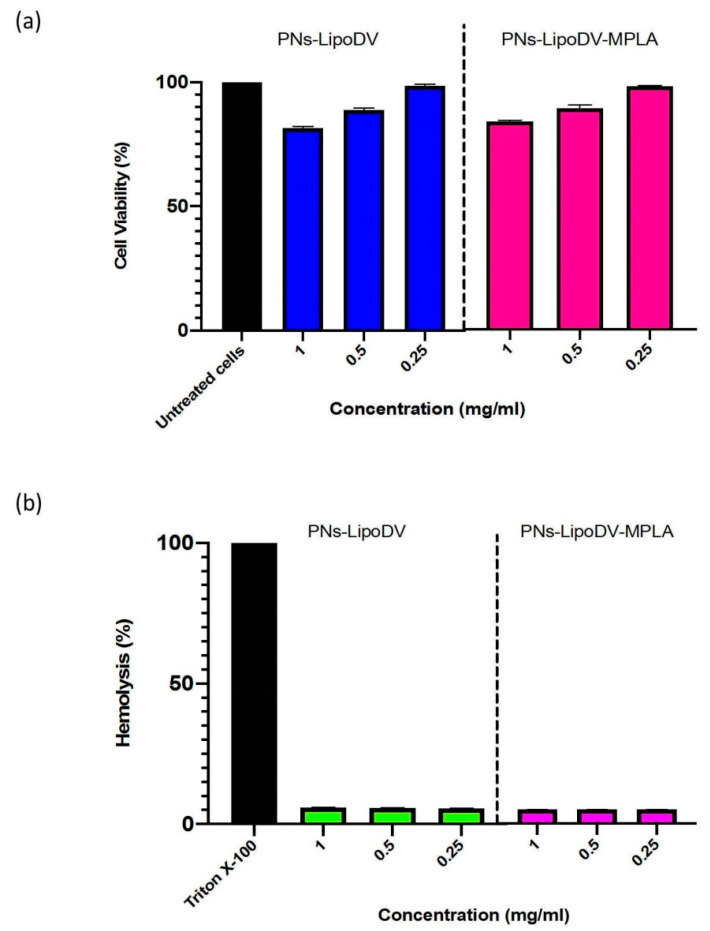
Cell viability analysis via MTT assay against HEK293 cells for 48 h as compared to the control cells (untreated cells, 100%) (**a**) and the hemolytic activity of formulated PNs vaccines at different concentrations range towards human red blood cells (**b**).

The cytotoxicity of PNs-LipoDV and PNs-LipoDV-MPLA vaccines was assessed on human embryonic kidney cells (HEK293) by MTT assay. PNs-based formulated vaccines concentrations were considered non-toxic when cell viability after 48 h of incubation remain above 80%. The MTT assay data demonstrated that both PNs-LipoDV and PNs-LipoDV-MPLA were non-toxic even at the highest tested concentration, 0.25 mg/mL (Figure 4a). The hemocompatibility of the formulated PNs vaccine was screened against human red blood cells. The in vitro hemolytic potential of the vaccine candidates was quantified as a relative percentage value compared to the detergent control (Triton-X) which induced 100% hemolysis. Our data demonstrated that both vaccines induced negligible hemolytic activity (≤6%) at the tested concentration ranges between 0.25 mg/mL to 1 mg/mL (Figure 4b). 

### 3.5. Antigen-Specific IgG Antibody Response to Dengue Vaccine Candidates 

**Figure 5:**
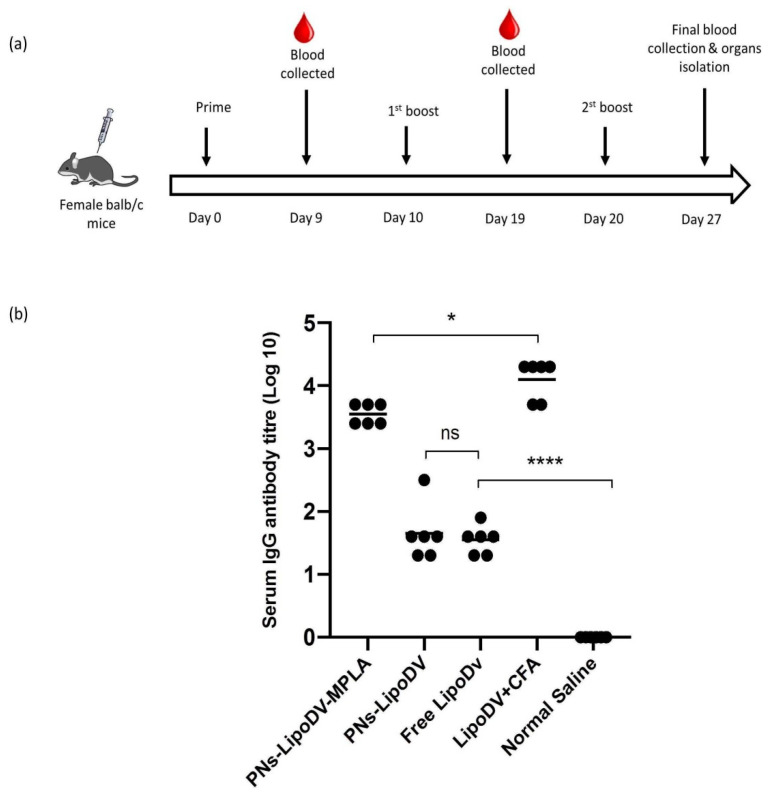
(**a**) Timeline of immunization and sample collection. Balb/c mice were subcutaneously immunized thrice in a 2-weeks interval. (**b**) Antigen-specific IgG antibody titers derived from the final blood collection sample after thrice vaccinations dose regime Each point in the figure represents an individual mouse in the group (n = 5). The mean antigen-specific IgG titers are represented as a bar. Statistical analysis was performed using one-way ANOVA followed by a post hoc test (Tukey) between the group as necessary. (ns, *p* > 0.05; * *p* < 0.05; **** *p* < 0.0001).

Next, we evaluated the immunogenicity effects of the polymersome-based DENV-mimicking nanoparticles vaccine, PNs-LipoDV-MPLA in the aspect of humoral immunity in mice following subcutaneous injections three times in 10 days interval (Figure 5a). The IgG response productions were compared with different vaccine candidates possessing respective systematic structural variations; PNs-LipoDV and free LipoDV. The antigen-specific IgG antibody titers of blood samples (final blood collection after the third boost) of immunized mice with the tested vaccine candidates, including positive control (LipoDV emulsified in CFA) and negative control (normal saline), were presented in Figure 5b. As expected, mice immunized with PNs-LipoDV-MPLA induced significantly higher IgG titers (*p* < 0.001) compared to PNs-LipoDV and LipoDV alone. There was no significant difference in IgG elicitation levels between mice immunized with PNs-LipoDV and free LipoDV. Interestingly, the IgG titers of PNs-LipoDV-MPLA was almost comparable to the positive control group, which was immunized with CFA-adjuvanted LipoDV, even though the antibody productions were relatively higher and statistically significant (*p* < 0.05).

### 3.6. The Th1/Th2 Cytokines Profiling of Vaccine Candidates

**Figure 6:**
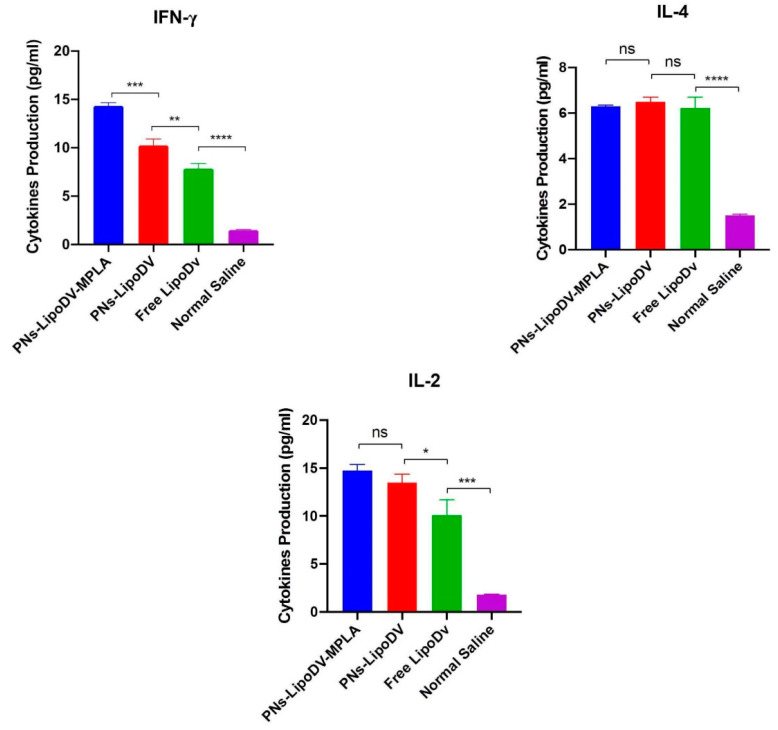
Splenocytes cytokines (IFN-γ, IL-2 and IL-4) response to immunization with vaccine candidates. Bars represent mean concentrations ± standard deviations. The level of cytokine secretions among the tested groups were compared using one-way ANOVA, followed by post hoc test (Tukey) (ns, *p* > 0.05; * *p* < 0.05; ** *p* < 0.01; *** *p* < 0.001; **** *p* < 0.0001).

The cytokines secretion of splenocytes from mice immunized by the vaccine candidates were assessed. Cytokines profiling were assessed based on Th1 favoring cytokines (IFN-γ, and IL-2) and the Th2-type cytokines favoring (IL-4). The Th1 and Th2 cytokines secretion in response to the tested vaccine candidates was shown in Figure 6. In general, all of the vaccine candidates induced significant levels of cytokines secretion compared to the negative control (mice immunized with normal saline) of the tested panel of cytokines The secretion of IFN-γ was significantly enhanced in mice immunized with PNs-LipoDV-MPLA compared to PNs-LipoDV and free LipoDV. For IL-2 and IL-4 secretions, there was no statistically significant difference between mice immunized with PNs-LipoDV-MPLA and PNs-LipoDV. Relatively, LipoDV formulated in polymersome nanoparticles platform have significantly induced a greater secretion of both IFN-γ and IL-2 cytokines compared to when administered alone. Nevertheless, in the case of IL-4 secretion, there was no difference between the tested vaccine candidates.

### 3.7. In Vivo Toxicity Evaluation 

The in vivo toxicity of the vaccine constructs was obtained by monitoring the sign of illnesses (including local reactions at the site of infections), death and weight loss of subsequent immunization of the tested vaccine candidates in mice. Based on physical observation, there was no death reported for the mice immunized with our vaccine candidates. Any symptoms of illness and apparent local reactions at the injection site (e.g., erythema and edema) were also negligible. Additionally, the changes of body weight in mice immunized with the tested vaccine candidates showed an increasing trend and were comparable to the mice group that was administered with normal saline (Supplementary Figure S2).

### 3.8. Stability Test of the Polymersome-Based Nanoparticles

The stability test of the fabricated PNs without the presence of vaccine antigen was tested up to seven months. Zeta potential, PDI and particle size of the blank PNs (with and without MPLA functionalization) were evaluated under different temperature conditions. The particle’s size and zeta potential of both PNs formulations (either with/without MPLA) increased after seven months (Supplementary Figure S3). However, the PDI value of PNs with MPLA coating remains comparable to its initial storage condition compared to PNs alone (greater than 0.3), indicating that the inclusion of MPLA enhanced polymersome stability and homogeneity for long-term storage.

### 3.9. Histological Examination of Liver and Kidney

**Figure 7:**
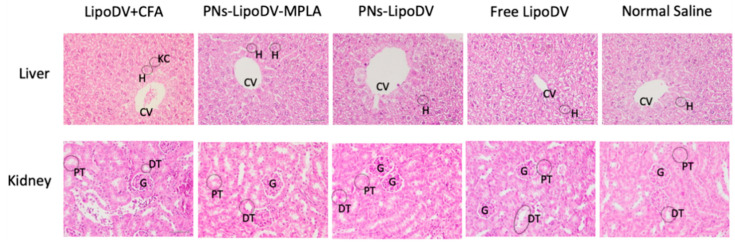
Histologic section of liver and kidney tissues of the immunized mice. Sections were stained with H&E and examined at 40× magnification. CV = central vein, KC = Kupffer cell, G = glomerulus, H = hepatocyte, PT = proximal convoluted tubules, DT = distal convoluted tubules.

Major secretion organs; liver and kidney were dissected out from the immunized mice (sacrifice on day 27), stained with H&E and were examined under a microscope within one week after harvest. As demonstrated in Figure 7, the histological examination of the liver samples of mice immunized with the tested vaccine candidates showed no morphological changes compared to mice treated with normal saline as a control group. However, for mice administered with the positive control (CFA-adjuvanted LipoDV), there was abnormal morphology observed in the liver samples that showed the presence of activated Kupffer cells and congestion of the central vein. Similarly, for kidney tissues, there were no histopathological changes were observed for mice immunized with all vaccine candidates when compared to the negative control group. In contrast to the histologic tissue samples of mice immunized with CFA-adjuvanted vaccine, suspected of cell proliferation of capsular epithelium due to induced nephrotic syndrome caused by CFA.

## 4. Discussion

Dengue is considered a serious and continuing threat to the global community, particularly in tropical and sub-tropical climates regions. There are no specific antiviral agents is currently available to treat dengue. The only licensed dengue vaccine, Dengvaxia is not clinically satisfactory due to lack of safety profile. Epidemiology evidence associates the use of Dengvaxia with ADE development in dengue-seronegative recipients [22]. Thus, the development of an effective dengue vaccine against all four different DENV serotypes and minimizing the risk of ADE development at the same time remain a challenge. Collective clinical and experimental data demonstrated that both neutralizing antibodies and T-cell oriented immune responses are necessary for vaccine-mediated protection against dengue [5].

In pursuit of addressing the problems, we have designed and constructed a multi-epitope peptide vaccine by fusing the B cell, Th and T cell epitopes in a single polypeptide chain bearing two copies of palmitic acid (LipoDV). The utilization of these minimal antigenic epitopes in vaccine design would enable the productions of highly targeted humoral and cellular immune responses, thus eliminating the risk of adverse reactions, including disease worsening condition via ADE mechanism. However, to be optimally effective, such peptide vaccines need to be administered with a potent vaccine delivery system. One of the attractive approaches in designing an efficient vaccine delivery system is by mirroring the morphology and pathogenic features of the targeted pathogen. Herein, we have formulated our novel nanoscale dengue vaccine delivery platform technology to mimic the structural features of DENV (Figure 1). LipoDV resembled the functionality of DENV E protein as the principal target for neutralizing antibodies induction and focal determinant for facilitating virus entry into the host cell membrane. LipoDV was immobilized in the hydrophobic region of the PBD-PEO polymersome nanoparticles that represent the DENV envelope. The PBD-PEO polymersome was functionalized with MPLA, an established immune-activating ligand that mimics the role of DENV M protein in maturing the virion particle during cellular infection. The DENV-mimicking nanoparticle vaccine formulation, PNs-LipoDV-MPLA was further tested for immunogenicity effect in mice, alongside PNs-LipoDV and free LipoDV for systematic structural variation comparison.

During the dehydration-rehydration process of PNs fabrication, LipoDV was solubilized alongside the PBD-PEO polymer to achieve its intercalation within the hydrophobic bilayer of the polymersome structure. It has been demonstrated that the localization of vaccine antigen on the surface of liposome bilayer induced better recognition by B cells compared to its entrapment inside the aqueous core [23]. The high EE of LipoDV may be contributed by the non-covalent hydrophobic interaction of the anchoring palmitic acids with the hydrophobic bilayers of polymersomes. Both PNs-LipoDV-MPLA and PNs-LipoDV were successfully fabricated with a uniform-size nanoscale dimension particle of approximately 130 nm as determined via DLS and TEM. The polymersome structure was visible and confirmed by TEM imaging (Figure 2). The additional incorporation of MPLA enhanced the stability of the polymersome as indicated by the increase in a negative value of zeta potential. It seems that MPLA exerts a similar impact to cholesterol in improving the stability of conventional liposome bilayer [24]. In the long-term stability study, both blank PNs (either with/without MPLA) retained acceptable structural integrity changes during the 7 months at all tested conditions, with PNs containing MPLA exhibiting a greater stability profile in terms of homogeneity distributions. Notably, at 40 °C, the particle size of NPs functionalized with MPLA was observed to be smaller while had an increased in PDI and negative value of zeta potential. This situation may be explained by the greater complexation between MPLA and the PBD_32_-PEO_21_ polymer which occurs over the time. The complexation may lead to a reduced particles size but affecting the uniformity of size distribution pattern.

The principal concept of incorporating vaccine antigens into nanoparticles is to promote their uptake by the APCs for efficient immune cells processing. The most targeted APCs in vaccine design are DCs and macrophages due to their central role in inducing the activation of the adaptive arm of the immune system. Our uptake analysis data showed that both nanovaccine formulations; PNs-LipoDV-MPLA and PNs-LipoDV were taken up efficiently by the studied APCs, although the uptake level was higher in DCs compared to macrophages (Figure 3). This may be attributed to the particle size of the PNs vaccines which was in the range of 120–130 nm. It was well established that DCs preferentially uptake virus-sized particles (20–200 nm) while macrophages take up more of the larger particles which is comparable to bacterial size (0.5–5 μm) [25]. In comparison to PNs-LipoDV, the uptake level of PNs-LipoDV-MPLA by DCs was statistically significantly reduced. The greater density of negative surface charge of PNs-LipoDV-MPLA may trigger a stronger repulsive force with the negatively charged cell membranes of DCs. A similar outcome on how the particle surface charge influences their uptake by APCs was also observed in other studies [26,27]. However, higher uptake by DCs does not warrant the initiation of immune responses [26]. A variety of other immune cascades, such as maturation of APCs, down-regulation of antigen internalization and surface expression of costimulatory molecules that govern the potentiation of immune response productions.

Therefore, the immunogenicity and adjuvanting potency of the vaccine candidates were evaluated in vivo. BALB/c mice were used in this study as they are highly responsive in inducing humoral immune response without compromising the activation of T-cell mediated immunity. The potent immunogenicity of our lead vaccine candidate, PNs-LipoDV-MPLA that was designed to mimic the structural morphology of DENV virion was reflected by the significantly higher antigen-specific antibody titers than PNs-LipoDV and free LipoDV (Figure 4b). However, the antibody productions of the mice group immunized with PNs-LipoDV-MPLA was comparatively lower than the positive group; LipoDV adjuvanted with CFA. CFA is regarded as the gold standard for adjuvants used in pre-clinical vaccine development; however, it is too toxic for clinical utility [13,28]. The toxicity of CFA was documented in the histopathology examinations of liver and kidney tissues obtained from mice immunized with CFA-adjuvanted antigen (Figure 7). The presence of activated Kupffer cells in the liver tissue indicated the formation of granulomas, which was highly associated with the administration of CFA [29]. Similarly, the mice group receiving CFA as vaccine adjuvant showed signs of nephrotic syndrome which was associated with CFA. In contrast to PNs-LipoDV-MPLA, there was no significant abnormal histopathological changes were observed in the examined organs as compared to the negative control.

Other than humoral immune response, the ability to induce cell-mediated immune response has been appraised as a critical attribute to DENV infection. Hence, we assessed whether our DENV-mimicking PNs-LipoDV-MPLA could demonstrate similar functional traits to DENV. The cytokine responses induced among tested vaccine candidates were evaluated. The polarity of the immune responses was monitored by quantifying the marker cytokines secreted by Th1 ((IFN-γ and IL-2) and Th2 (IL-4) cells. The results demonstrated that the secretion of IFN-γ was significantly elevated in splenocytes from mice vaccinated with PNs-LipoDV-MPLA in comparison to PNs-LipoDV and free LipoDV, while the secretion of IL-2 and IL-4 were comparable among the tested groups (Figure 5). The obtained data indicated that our designated vaccine antigen, LipoDV possess self-adjuvating properties. The strategy in anchoring two copies of palmitic acid (C16) to the multi-epitope peptide antigen significantly promoted humoral immune response induction as indicated by high-level IL-4 cytokines secretion, as well as the antigen-specific antibodies productions. The self-adjuvanting property of lipopeptides as vaccine antigen was well observed in various studies [13,30,31,32]. It was documented that the induction of IFN-γ is essential for host reaction in conferring substantial protection against DENV virus infection [33]. As the level of IFN-γ secretion was the highest in mice immunized with PNs-LipoDV-MPLA, our strategy in utilizing MPLA as immunostimulatory molecules enhanced the polarization of the Th1 cell. The adjuvanting property of MPLA was primarily biased towards the induction of cell-mediated immune response as reported in previous studies [34,35,36]. Thus, our collective data demonstrated that immunization with PNs-LipoDV-MPLA induced a potent humoral and cell-mediated immune response with enhanced IFN-γ secretions which emulated DENV infections.

Importantly, all the tested vaccine candidates, especially the lead formulation (PNs-LipoDV-MPLA) were found to be non-toxic to the normal cells and did not induce hemolysis. Additionally, the in vivo toxicity evaluations showed that no symptoms of illness (including body weight changes), local reaction at the injection site and death were observed in mice immunized with our formulated dengue vaccines, thus confirming their safety.

## 5. Conclusions

In summary, we have successfully constructed a DENV-mimicking vaccine delivery platform (PNs-LipoDV-MPLA) based on polymersome nanoparticles intercalated with lipidated antigenic polypeptide targeting B and T cells receptor activation and functionalized with immunostimulatory MPLA on the polymersome surface. The cellular uptake studies demonstrated that PNs-LipoDV-MPLA possessed a favorable nanoscale dimension for efficient uptake by the DCs. Comparative immunization studies in mice showed that the DENV-mimicking vaccine PNs evoked a stronger humoral immune response with an enhanced Th1 activity than its relative vaccine constructs with lesser structural functionality of DENV. PNs-LipoDV-MPLA did not exhibit any cytotoxicity effects even tested at high dose against normal cells as demonstrated by MTT and hemolytic assay. Finally, in vivo toxicity evaluation including the histopathological examination of the major organs for metabolism dissected from the immunized mice indicated the excellent safety profile posed by PNs-LipoDV-MPLA. Thereby, the collective experimental data indicated that our strategy in fabricating peptide-based vaccine delivery platform which resembled the morphology and structural functionalities of DENV hold a great promise for future development of a safer and efficacious dengue vaccine.

## Figures and Tables

**Table 1:** Physicochemical characterizations and antigen encapsulation efficiency of PNs-based vaccine.

Formulations	Particle Size (nm)	PDI	Zeta Potential (mV)	EE (%)
PNs-LipoDV	130.73 ± 0.90	0.205 ± 0.01	−11.37 ± 0.35	94.19
PNs-LipoDV-MPLA	129.67 ± 0.40	0.204 ± 0.00	−17.13 ± 0.40	93.71

## Data Availability

The data presented in this study can be made available upon request from the corresponding author.

## Supplementary Materials

The following are available online at https://www.mdpi.com/article/10.3390/pharmaceutics14010156/s1, Figure S1: Cell populations from flow cytometry analysis of the dendritic cells and macrophage following incubation with PNs-based nanoparticles, Figure S2: The average body weight changes of mice after immunization with the tested vaccine candidates, Figure S3: The stability of blank polymersome nanoparticle formulations.
