# Supplementary material for: DENV-Mimetic Polymersome Nanoparticles Bearing Multi-Epitope Lipopeptides Antigen as the Next-Generation Dengue Vaccine
Source: Pharmaceutics. 2022 Jan 10;14(1):156. doi: 10.3390/pharmaceutics14010156 (PMC8781246; doi:10.3390/pharmaceutics14010156)
Supplement: Supplementary file 1 [file pharmaceutics-14-00156-s001.zip › pharmaceutics-1529707-supplementary.pdf]

pharmaceutics

Supplementary materials: DENV-Mimetic Polymersome Nanoparticles Bearing Multi-Epitope Lipopeptides Antigen as the Next-Generation Dengue Vaccine

Nur Adilah Abdul Rahman, Abdin Shakirin Mohamad Norpi, Muhammad Luqman Nordin, Mohd Cairul Iqbal Mohd Amin, Abdullah Al-Hadi Ahmad Fuaad, Nor Azila Muhammad Azami, Nirmal Marasini and Fazren Azmi

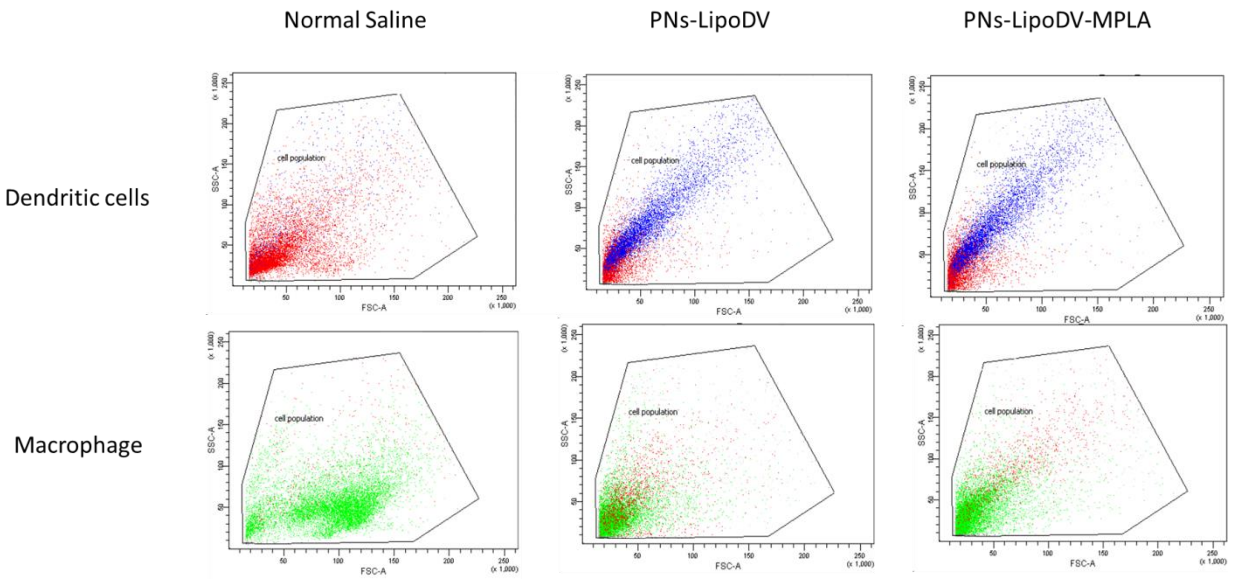

Figure S1. Cell populations from flow cytometry analysis of the dendritic cells and macrophage following incubation with PNs-based nanoparticles.

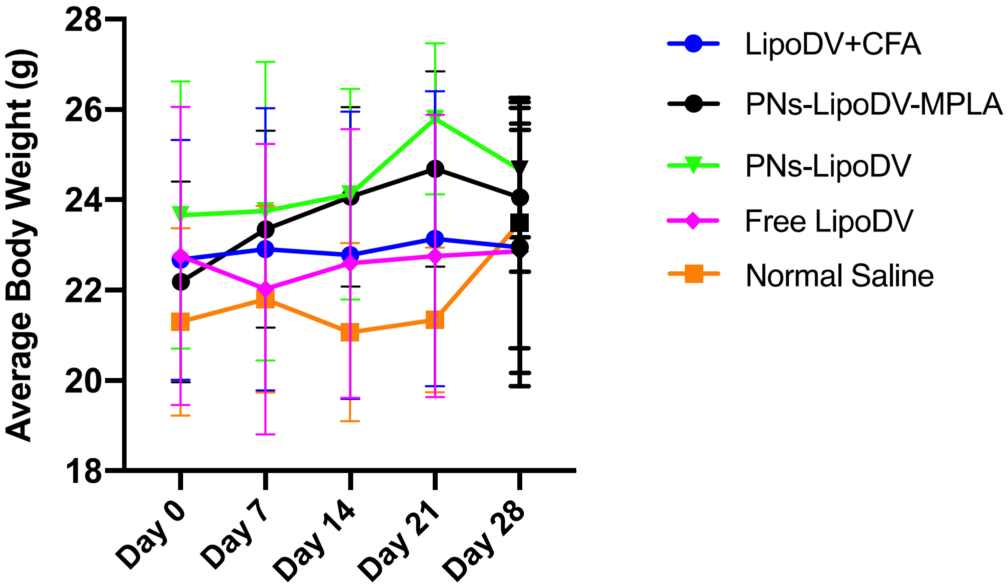

Figure S2. The average body weight changes of mice before and after immunization with the tested vaccine candidates.

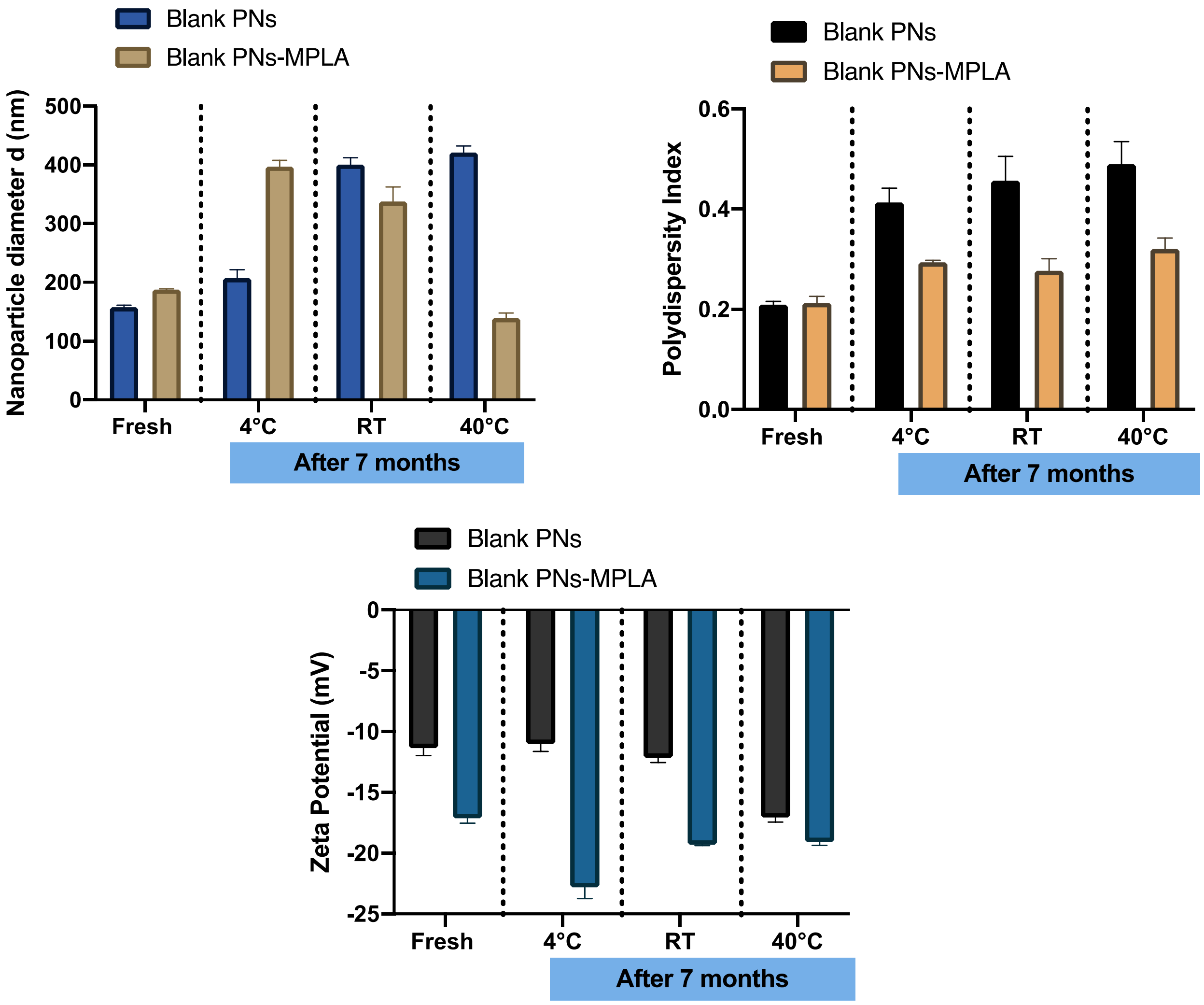

Figure S3. The stability of blank polymersome nanoparticle formulations (with and without the presence of MPLA coating). The particle's size diameter, PDI and zeta potential were measured after 7 months under different temperature conditions (4 °C, room temperature and 40 °C).
